# Unmasking of a large pheochromocytoma by pyelonephritis

**DOI:** 10.1002/ccr3.2464

**Published:** 2019-09-30

**Authors:** Igor Alexander Harsch, Herry Helfritzsch

**Affiliations:** ^1^ Division of Endocrinology and Metabolism Department of Internal Medicine II Thuringia Clinic Saalfeld “Georgius Agricola” Saalfeld/Saale Germany; ^2^ Department of General, Visceral and Thoracic Surgery Thuringia Clinic Saalfeld “Georgius Agricola” Saalfeld/Saale Germany

**Keywords:** hypertension, pheochromocytoma, pyelonephritis

## Abstract

Pheochromocytoma is a rare reason for hypertension. At least in younger people, an endocrinological workup of the etiology of hypertension is mandatory, as this case describes. If the pheochromocytomas are large enough, they can not only lead to hypertension but also cause direct damage to surrounding organs.

## Graphical abstract

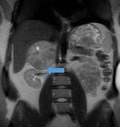

## 1 CASE REPORT

The case of a young patient with long‐standing hypertension is described. Unfortunately, no endocrinological assessment had been performed in the past. The diagnosis of pheochromocytoma was established when a pyelonephritis of the right kidney occurred, presumably due to compression effects.

**Figure 1:**
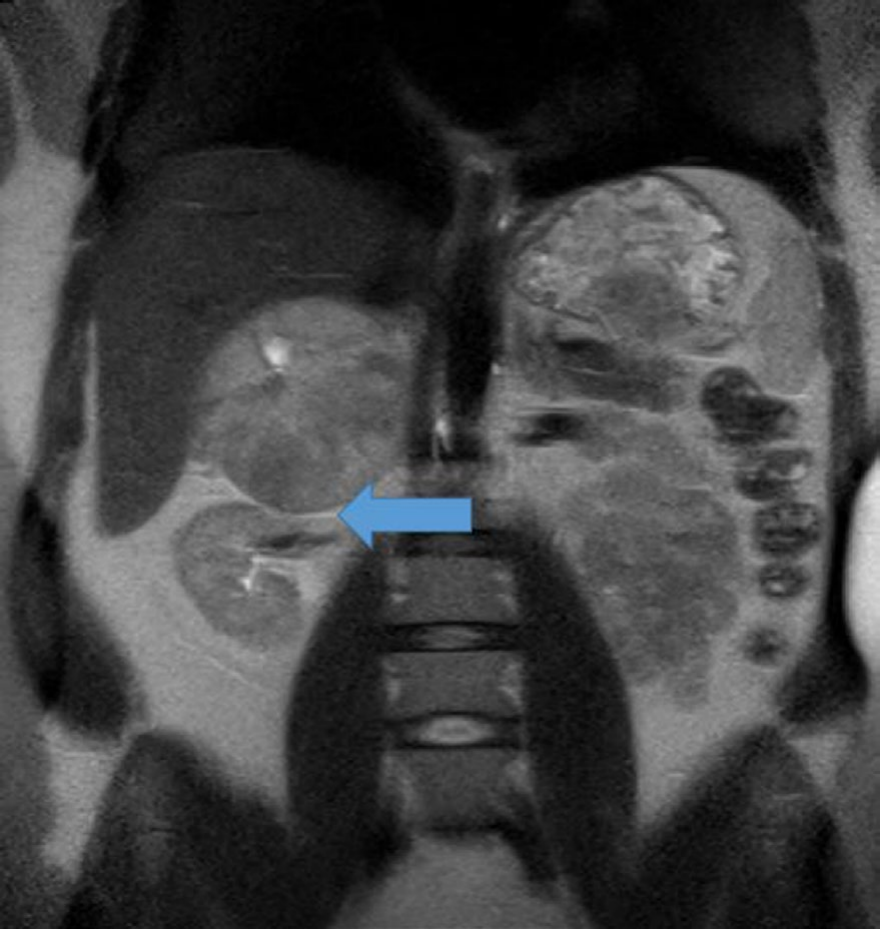
The magnetic resonance imaging shows the pheochromocytoma with a size of about 9.7 × 6.9 cm and good demarcation to the surrounding tissue (above arrow). The inferior vena cava is slightly compressed, as is the kidney (see arrow)

A 31‐year‐old female patient had feverish pyelonephritis. Further workup revealed a 10‐cm right adrenal mass by MRI (Figure 1). Serum metanephrine and normetanephrine levels after stopping antihypertensives were significantly elevated (1210 ng/l [<90] and 16 050 ng/l [<200]). The patient had previously been treated with several antihypertensive drug classes since the age of five. Hypertensive retinopathy was known. Systolic blood pressure was recorded in a range 140‐160 mm Hg here. Hypertensive crises had never occurred. After adequate alpha‐ and beta‐blockade with doxazosin and later propranolol for 5 weeks (alpha‐receptors are widely distributed in the nasal mucosa: a congested nose is a valuable clinical sign of adequate alpha blockade), she underwent surgery. Histology revealed a “Pheochromocytoma of the Adrenal gland Scaled Score” (PASS) score of 4 thus indicative of a low aggressive potential.

**Figure 2:**
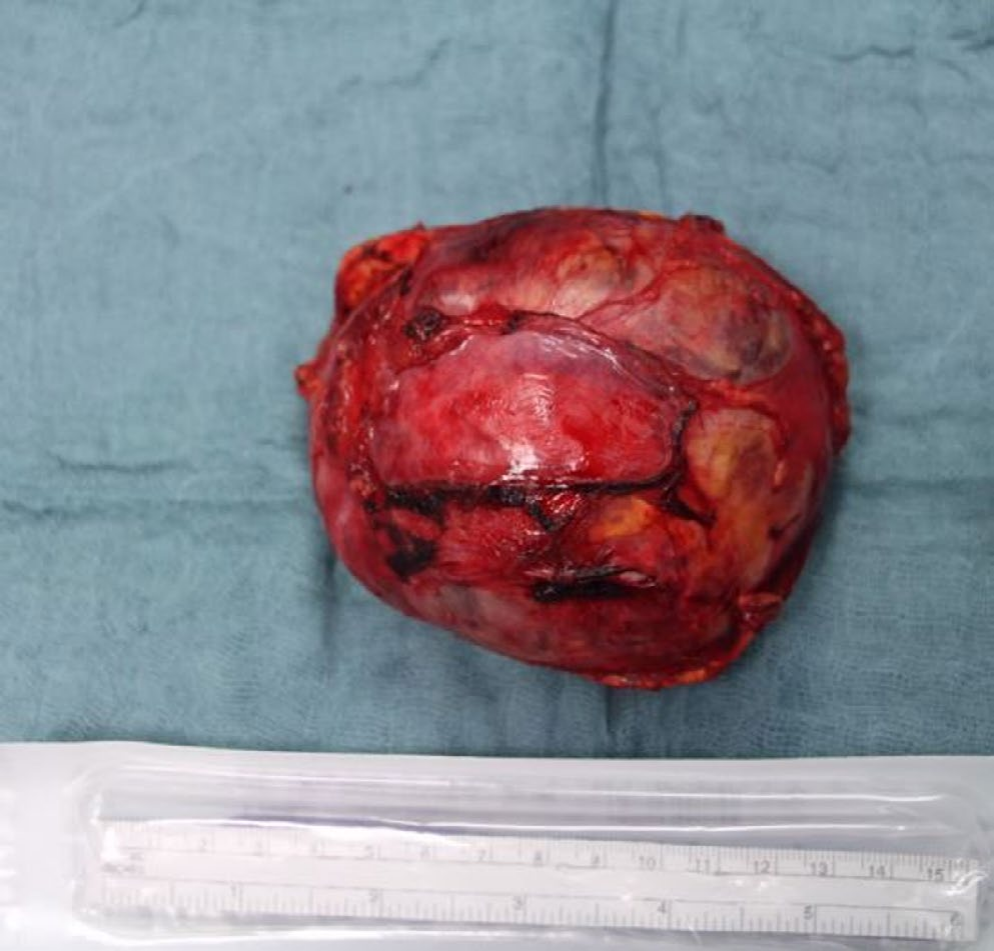
Surgical preparation of the completely removed pheochromocytoma. Weight: 272 g

The pheochromocytoma was larger than the average size (Figure 2) reported in the literature (3‐5 cm).^1^ It can be speculated that pyelonephritis occurred as a result of a renal compression 2 or disturbance in renal blood flow. Chronic long‐standing compression of the kidney can also result in hypertension (“Page kidney”): Decreased blood flow to the kidney triggers excess secretion of renin. This phenomenon may contribute to hypertension here—10 months after surgery, the patient is still treated with ramipril. Our observations underline the importance of an endocrinological checkup of hypertension, especially in young patients.

## CONFLICT OF INTEREST

There are no conflicts of interest.

## AUTHOR CONTRIBUTIONS

IAH: wrote the article and has accountability for all aspects of the work. H.H: performed the surgery and contributed to the text.

## ETHICAL APPROVAL

The patient gave written consent to report her case and the imaging.
